# Breast Reconstruction–Prospective Follow up on Breast Cancer Patients’ Health-Related Quality of Life

**DOI:** 10.1007/s00268-021-06426-4

**Published:** 2022-01-09

**Authors:** Mervi Rautalin, Tiina Jahkola, Risto P. Roine

**Affiliations:** 1 Department of Plastic Surgery, Helsinki University Hospital and University of Helsinki, Töölö Hospital, Topeliuksenkatu 5, 00029 HUS Helsinki, Finland; 2 Department of Plastic Surgery, Helsinki University Hospital, Helsinki, Finland; 3 Department of Health and Social Management, University of Eastern Finland, Kuopio, Finland

## Abstract

**Background:**

Analysing the results of breast reconstruction is important both in terms of oncological safety and health-related quality of life (HRQoL). Immediate breast reconstruction (IBR) is thought to be prone to complications and heavy for patients with no time to adapt to having cancer. Delayed reconstruction (DR) is an option after primary surgery and oncological treatments, but requires patients to go through two recovery periods after surgery.

**Methods:**

A prospective study of 1065 breast cancer patients with repeated measurement of HRQoL with both generic (15D) and disease specific (EORTC QLQ C-30 BR23) measuring tools included 51 IBR patients and 41 DR patients. These patients’ HRQoL and reconstruction methods were studied in more detail alongside with clinical data to determine HRQoL levels for patients with IBR and those with mastectomy and DR during a 24-month follow-up. Measuring points were baseline, 3, 6, 12 and 24 months.

**Results:**

Most frequent techniques used were abdominal flaps (IBR *n* = 16, DR *n* = 14), latissimus dorsi flaps (LD) (IBR *n* = 19, DR *n* = 10), implants (IBR *n* = 12) and fat grafting (DR *n* = 6). Smaller groups were excluded from group comparisons. Approximately one third of the patients encountered complications. Symptom scores did not differ between reconstruction methods. DR patients had better overall HRQoL at 12 months, but at 24 months the situation had changed in favour of IBR. Both approaches of reconstructive surgery produced good HRQoL with no significant differences between the approaches studied.

## Introduction

### Background

Breast cancer surgery has three options: breast conserving surgery, mastectomy, or reconstruction. The surgical approach has evolved in time as the evidence on oncological safety has built up [1, 2]. Breast conserving surgery combined with radiation therapy is considered as the first treatment option both in terms of producing an oncologically safe procedure and good patient satisfaction [3]. If breast conserving surgery is not applicable, breast reconstruction is an option. Breast reconstruction improves health-related quality of life (HRQoL) after the initial shock of having cancer and going through cancer treatments [4].

The breast can be reconstructed at the first operation (immediate reconstruction, IBR) or after primary surgery and oncological treatments (delayed reconstruction, DR). Individual surgical treatment is tailored for each patient according to national guidelines combining all aspects that affect the treatment options; overall health status of the patient, the characteristics of the breast tumour, and the size of the tumour and/or the breast. Patient’s own wish is part of the decision making in collaboration with the surgeon [5, 6].

The most favourable timing of breast reconstruction is yet under debate as is also the best reconstruction method. The nature of breast reconstruction is a slow process and HRQoL data on these patients are still scarce. Long operating times or complications associated with demanding reconstructive surgery may influence HRQoL [7]. In past studies, the recovery time after cancer treatments has been considered to be about one year. Thus, the natural impairment of HRQoL after diagnosis and treatment may be long lasting until HRQoL eventually reaches the level prior to cancer diagnosis. Studies on HRQoL and reconstruction have often been retrospective or focussed on limited surgical methods [8, 9]. Some HRQoL instruments have high ceiling effects and thus are not applicable for this patient group. By contrast, the overall health status is not well presented with all disease specific instruments. Therefore, prospective studies are needed to explore both allogenous and autologous reconstruction methods and use of both disease specific and generic HRQoL instruments [10–14].

### Objectives

The objective of this study is to describe the healing process of breast cancer patients with breast reconstruction. We investigated the HRQoL of IBR patients and the HRQoL of patients who undergo first cancer surgery (mastectomy) and then go through reconstruction (DR) during a 24-month follow-up period. Furthermore, we investigated if there are differences between different reconstruction methods and does this reflect on HRQoL.

## Material and methods

### Patient characteristics

This study was approved by University of Helsinki Ethics committee §68 (11.6.2008, 207/13/03/02/08). The recruitment process, with a goal of gathering 1000 + patients, lasted from September 2008 to September 2015 at the breast cancer unit of the Comprehensive Cancer centre of the Helsinki University Hospital. Patients were asked to participate at the first visit prior to surgery and later approached via mail. Clinical data were collected from hospital records and analysed in association with the HRQoL questionnaires. IBR was performed for 51 patients. Within the 24-month follow-up, an additional 41 patients were identified, who had had corrective surgery after oncological treatments and thus formed our DR group (Table 1). Comorbidities were identified from patient records with ICD-10 codes and rated using the Charlson Comorbidity Index score system [15, 16].

**Table 1:** Patient characteristics

	Immediate					Delayed					
Reconstruction type, *n*	Total 51	Abdominal 16	LD 19	Implant 12	TMG 4	Total 41 (1 LAP)	Abdominal 14	LD 10	Implant 3	Fat graft 6	Reduction 7
Age at time of reconstruction, mean	48.5 (25–64)	48.4 (25–63)	53 (32–64)	44 (28–62)	41 (27–56)	53.9 (26–79)	52.7 (36–65)	55.3 (41–75)	47 (33–55)	48 (34–55)	61 (26–79)
BMI at baseline, mean	25.2	26.3	25.5	23.6	23.9	24.6	25.1	24.4	21.6	22.5	27.4
Months from primary surgery, mean (range)	–	–	–	–	–	20.5 (10–24)	20.6 (12–24)	21.1 (15–24)	24	22 (19–24)	16.3 (10–23)
Axillary clearance, n (%)	19 (37.3)	7 (43.8)	7 (36.8)	4 (33.3)	1 (25)	20 (48.8)	5 (35.7)	4 (40)	3 (100)	2 (33.3)	5 (71.4)
Ductal carcinoma, *n* (%)	27 (52.9)	7 (43.8)	10 (52.6)	8 (66.7)	2 (50)	27 (65.9)	9 (64.3)	5 (50)	1 (33.3)	6 (100)	5 (71.4)
Lobular carcinoma, *n* (%)	13 (25.5)	5 (31.3)	5 (26.3)	2 (16.7)	1(25)	11 (26.8)	3(21.4)	5 (50)	2 (66.7)	0	1(14.3)
DCIS, *n* (%)	5 (9.8)	1 (6.3)	3 (15.8)	0	1 (25)	0	0	0	0	0	0
Stage 1a/1b *n*(%)	20(39.2)/0	6(11.8)/0	5(9.8)/0	7(13.7)/0	2(3.9)/0	16(39)/0	6(14.6)/0	6(14.6)/0	1(2.4)/0	1(2.4)/0	2(4.9)/0
Stage 2a/2b *n*(%)	10(19.6)/14(27.5)	6(11.8)/2(3.9)	1(2)/11(21.6)	2(3.9)/1(2)	1(2)/0	19(46.3)/4(9.8)	7(17.1)/1(2.4)	3(7.3)/1(2.4)	1(2.4)/0	5(12.2)/0	3(7.3)/2(4.9)
Stage 3a/3b *n*(%)	6(11.8)/0	2(3.9)/0	1(2)/0	2(3.9)/0	1(2)/0	0/0	0/0	0/0	1(2.4)/0	0/0	0/0
Radiation therapy, *n* (%)	17 (33.3)	4 (25)	6 (31.6)	5 (41.7)	2 (50)	22 (53.7)	7 (50)	4(40)	1 (33.3)	4 (66.7)	5 (71.4)
Chemotherapy, *n* (%)	32 (62.7)	10 (62.5)	8 (42.1)	11 (91.7)	3 (75)	26 (63.4)	7 (50)	7 (70)	2 (66.7)	4 (66.7)	5 (71.4)
Endocrine treatment, *n* (%)	34 (66.7)	13 (81.3)	12 (63.2)	7 (58.3)	2 (50)	35 (85.4)	12 (85.7)	9 (90)	2 (66.7)	6 (100)	5 (71.4)
Targeted therapy, *n* (%)	7 (13.7)	0	1 (5.3)	5 (41.7)	1 (25)	8 (19.5)	3 (21.4)	1 (10)	1 (33.3)	1 (16.7)	2 (28.6)
Recorded complication, *n* (%)	18 (35.3)	4 (25)	8 (42.1)	6 (50)	0	13 (31.7)	7 (50)	3 (30)	0	0	2 (28.6)
Length of stay at reconstruction phase, mean days (range)	5.4 (2–9)	6.5 (2–9)	5.8 (2–7)	3.1 (2–6)	6.5 (6–7)	4.7 (1–14)	7.1(5–10)	5.2 (4–7)	3(2–4)	1.3 (1–2)	1.7 (1–2)
Smoking, active, *n* (%)	5 (9.8)	2 (12.5)	3 (15.8)	0	0	3 (7.3)	2 (14.3)	1 (10)	0	0	0
Smoking, quitted, *n* (%)	8 (15.7)	3 (18.8)	2 (10.5)	2 (16.7)	1 (25)	3 (7.3)	1 (7.1)	2 (20)	0	0	0

### Complications

Complications were recorded from patient files and classified according to the Clavien-Dindo classification for complications (Class I = deviations from normal recovering course with no need for revision or antibiotics; Class II = deviations from normal recovering course with a need for revision or antibiotics; Class IIIa = complications requiring intervention with local anesthesia; Class IIIb = complications requiring intervention with general anesthesia; Class IV = life-threatening complications; Class V = death) [17, 18].

### HRQoL questionnaires

HRQoL questionnaires used in our study were the generic 15D and the disease specific EORTC QLQ C-30 BR-23. Patients signed an informed consent form and filled in the questionnaires five times: at baseline and 3, 6, 12 and 24 months after start of treatment.

### 15D

The 15D is a validated generic HRQoL instrument, which addresses HRQoL with 15 questions concerning: moving, seeing, hearing, breathing, sleeping, eating, speaking, excretion, usual activities, mental functioning, discomfort and symptoms, depression, distress, vitality and sexual functioning. It also produces a total HRQoL score. Patients answer the questions concerning all dimensions by choosing a value from 1 (best) to 5 (worst situation) [19, 20].

### EORTC QLQ C-30 BR23

The EORTC QLQ C-30 is widely acknowledged and used HRQoL instrument. It is cancer specific and the additional BR23 is especially designed for breast cancer patients. This tool produces functional scores: physical, emotional, social, cognitive, role and with BR23 body image, sexual functioning, sexual enjoyment and future perspective functioning. It also addresses directly symptom scores; pain, fatigue, sleeping disturbances, constipation, diarrhoea, appetite loss and BR23 specifically systemic therapy side effects, breast and arm symptoms and upset by hair loss. Functional score questions are rated from 1 to 4, where a high score indicates good functioning in that area. On the contrary, high symptom scores indicate problems in that area. Patients rate symptom- questions from 1 to 7 [21, 22].

### Statistical analysis

Analysis was performed with NCSS software. To ensure the normal distribution we did the Box-Cox transformation for the data. We performed Two-Sample tests where the grouping variable was patients with immediate reconstruction (*n* = 51) or delayed reconstruction (*n* = 41). We compared the groups’ overall quality of life (15D score and EORTC global health score) and studied the change in those from baseline to 12 months and 24 months. The Mann–Whitney U-test was used for comparing the two group’s BR-23 answers, EORTC symptom and functional scores and 15D health profiles. Correlation was studied between overall quality of life scores and comorbidities, stage of the disease (Spearman correlation); complications, recurrences and having to go through more than one operation (Mann–Whitney U-test).

To study the difference between the major reconstruction group’s symptom and functional scores at 12 and 24 months, the analysis was done with Kruskal–Wallis nonparametric ANOVA. Because of the group sizes, the major IBR reconstruction methods that were possible to compare were abdominal flap, latissimus dorsi (LD) flap and implant patients. For DR the groups were abdominal flap, LD flap and fat grafting. Other groups were discarded as being too small in group size or clinically non-important when studying the difference between reconstruction methods (IBR 4 transverse musculocutaneus gracilis flaps (TMG) and DR reduction of contralateral side, 3 implant and 1 lumbar artery perforator (LAP) flap).

## Results

### Response rate and missing answers

Those willing to participate were meticulous in returning the questionnaires. The percentages for response rates were from baseline to 3 months (IBR/DR) 94.1/92.7%, 6 months 98/100%, 12 months 98/95.1% and 24 months 92.2/92.7%. There was missing data for only 1 patient’s answers in the IBR group at 12 months and 1 patient’s answers in the DR group at 24 months.

### Immediate breast reconstruction

IBR was performed for 51 patients. Three of them had had an earlier breast cancer. 19 patients were reconstructed with LD flap; 10 had an implant in addition to the flap, 16 were reconstructed with an abdominal flap, 12 with implant only and 4 with TMG. Abdominal flaps were 9 TRAM (transverse rectus abdominis muscle flap) and 7 DIEP (deep inferior epigastric perforator flap). All implants were expanders inducing a second operation where a permanent implant was placed. 19 patients had axillary clearance, the rest had sentinel node biopsy (snb). One patient had Stage 0, the rest as presented in Table1.

IBR patients’ mean age was 48.5 years, SD 11.0 (range 25–64). Comorbidities were rare: 42 had a Charlson comorbidity index rating of 0, 4 had 1, 4 had 2, and 1 patient had a 3-point rating. Smoking status was active for 5 patients and 8 had a history of smoking. Mean Body-mass index (BMI) was 25.2 (range 18.1–35.2).

Oncological treatments were radiation therapy for 17, chemotherapy for 32, endocrine treatment for 34 and targeted therapy (Herceptin) for 7 patients (Table 1).

One IBR patient with implant and snb had a recurrence (ductal grade 3 T2 disease). At 16 months she presented axillary metastases resulting in axillary clearance.

### Delayed reconstruction

Delayed reconstruction was performed for 41 patients within the 24-month study period. Balancing reduction or mastectomy on contralateral (healthy) side was done for 7, abdominal flap for 14, LD flap for 10, implant for 3, LAP for 1, and fat grafting for 6 patients. 6 abdominal flaps were TRAM and 8 DIEP.

DR patients’ mean age was 53.9 years, SD 10.7 (range 26–79) at time of reconstruction. Mean time from primary cancer surgery, i.e. the time of reconstruction from baseline was 20.6 months (range 12–24). Active smoking status was found for 3 patients, 3 had a history of smoking. Axillary clearance was performed for 20 patients during primary surgery, the rest had snb. Majority of patients had no comorbidities; 3 had 1 and 4 had 2 comorbidities. Mean BMI was 24.6 (range 19.8–33.5) (Table 1).

One DR patient had a recurrence at 1 year follow up on contralateral side resulting to bilateral DIEP reconstruction.

### Overall HRQoL

**Fig. 1:**
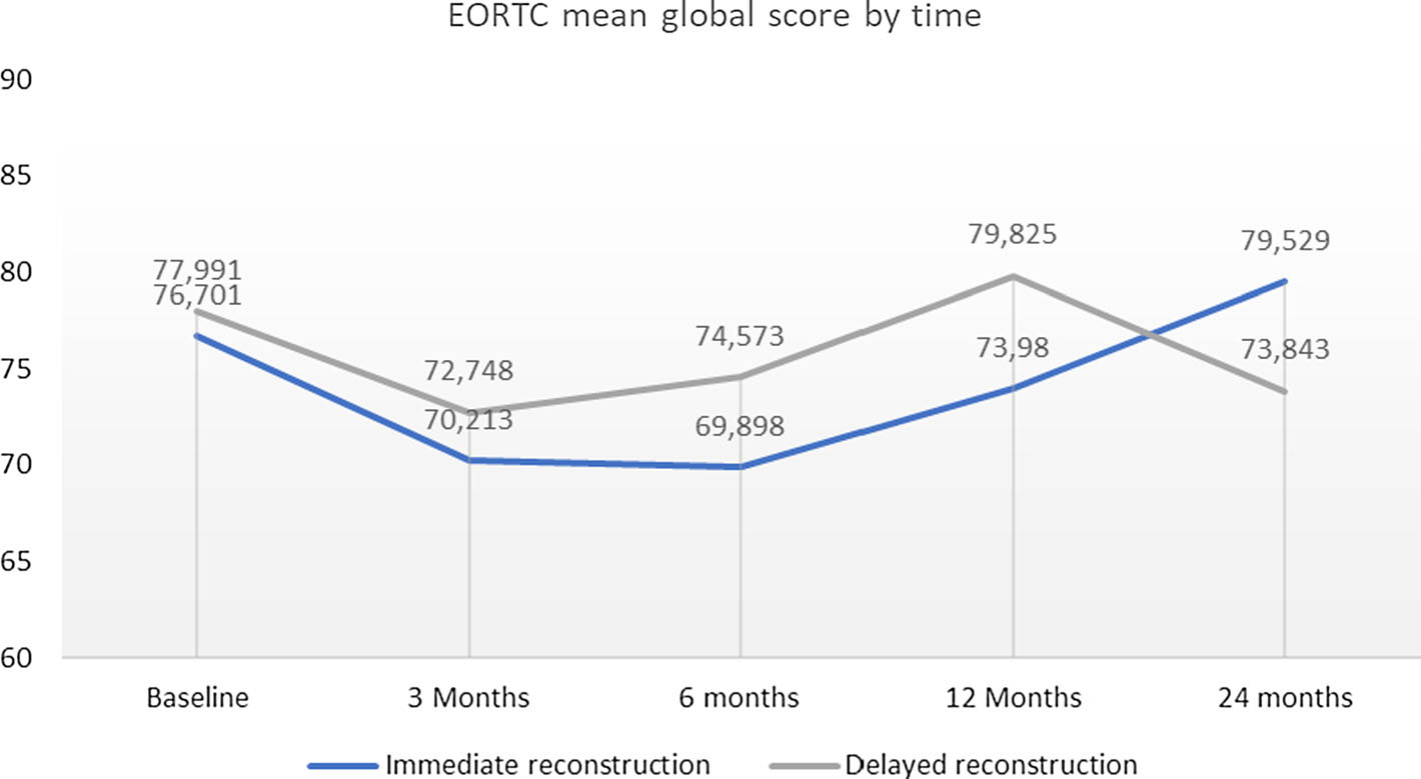
EORTC mean global score by time for Immediate and Delayed reconstruction groups

There was a statistically significant difference between the IBR and DR groups in the change of the EORTC global score from 12 to 24 months (*p* = 0.017). In IBR patients, HRQoL started to rise after 12 months whereas in DR patients it diminished. At 24 months, IBR patients obtained a higher EORTC global score than at baseline, whereas in DR patients it was lower (Fig. 1).

**Fig. 2:**
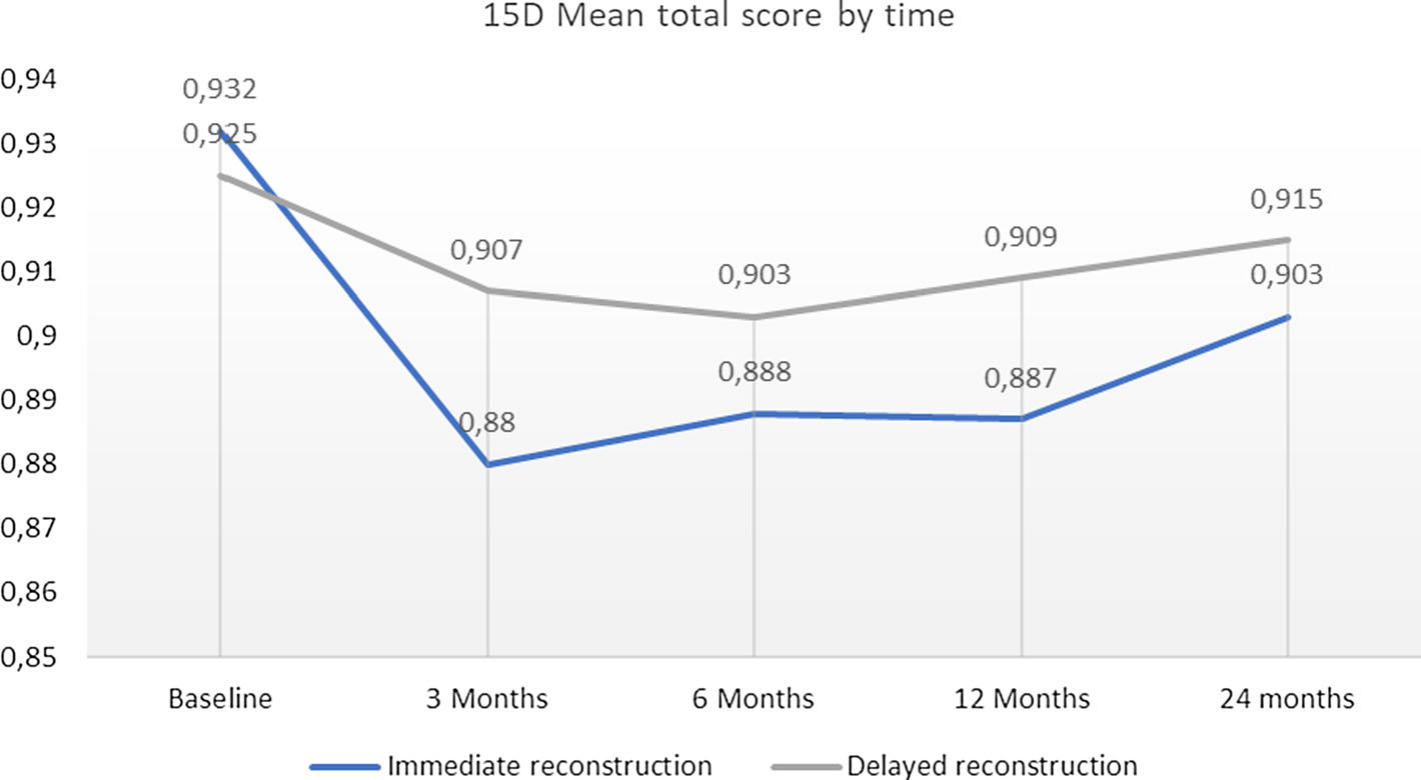
15D mean total score by time for Immediate and Delayed reconstruction groups

With the 15D, the difference between the 2 groups was non-significant when comparing the change in overall HRQoL from 12 to 24 months. In both groups the 15D score improved after an initial drop, but neither group reached at 24 months the same level than at baseline (Fig. 2).

**Fig. 3:**
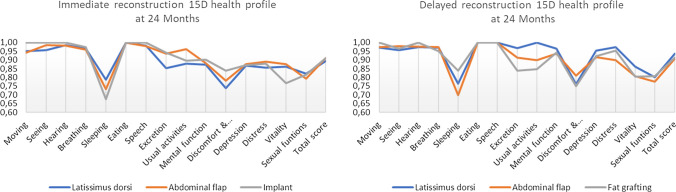
15D health profiles for Immediate and Delayed reconstruction patients at 24 months. Mean score values for different surgical methods in main groups

**Fig. 4:**
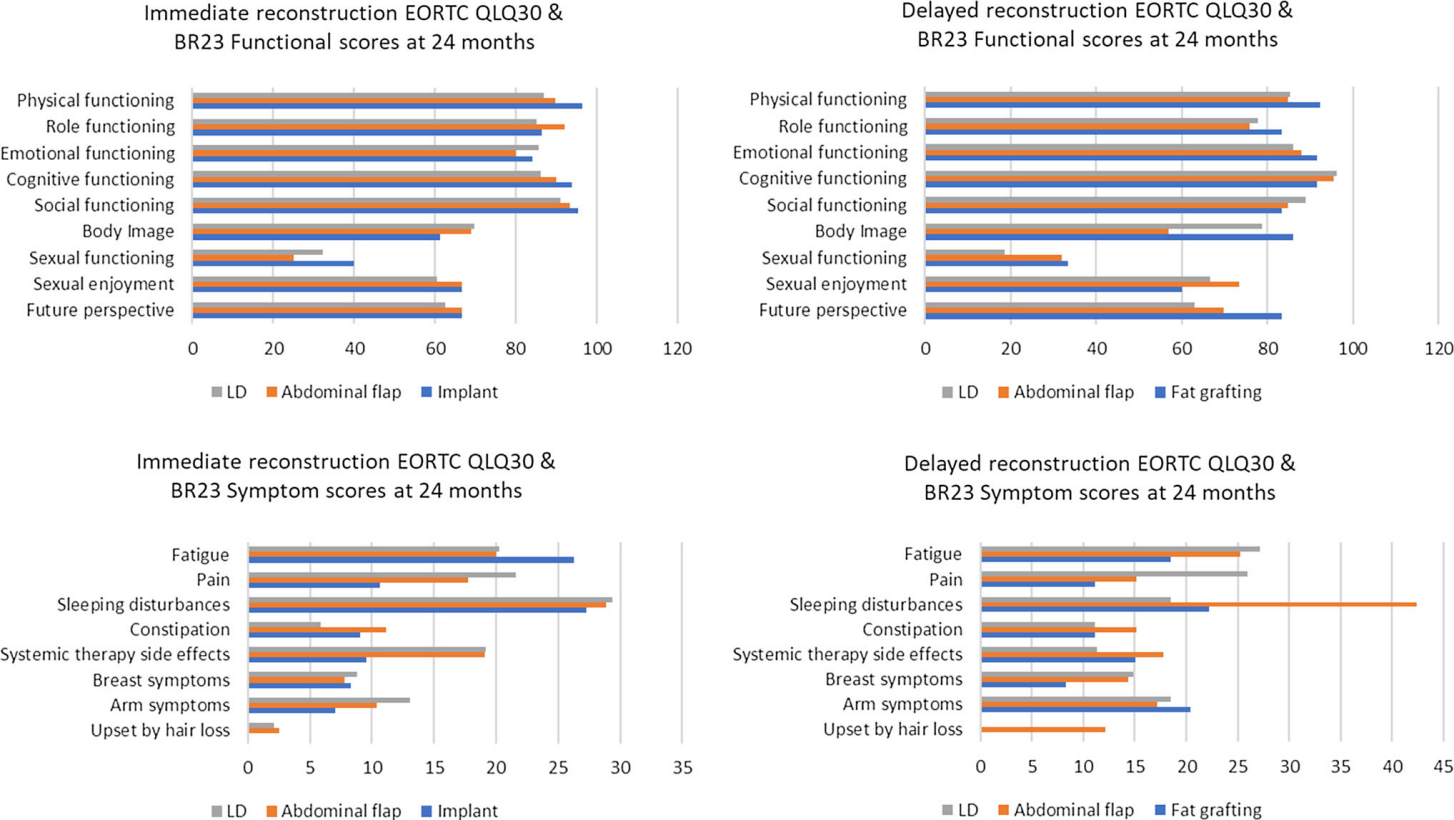
EORTC QLQ30 and BR23 functional and symptom scores mean values for immediate and delayed reconstruction at 24 months for different surgical methods

In the IBR group there was no difference between the operation methods in overall HRQoL at 12 months (15D *p* = 0.82 and EORTC *p* = 0.98) or at 24 months (15D *p* = 0.91 and EORTC *p* = 0.59). The same was true for DR patients as the overall HRQoL did not differ between operation methods with either tool (15D *p* = 0.76 and EORTC *p* = 0.47 (Figs. 3 and 4).

### The effect of patient characteristics on HRQoL

Both IBR and DR patients had little comorbidities, which didn’t affect HRQoL. High disease burden (Stage) correlated with EORTC at 24 months for IBR, *p* = 0.0253, but for DR patients no correlation was found. Axillary clearance, having to go through more than one operation or encountering a recurrence did not affect the HRQoL at 24 months (*p* > 0.05).

### HRQoL dimensions, symptom—and functional scores

#### The change of scores

To study whether the groups behaved in a similar way we analysed the change in the scores in relation to time. We compared the change of reported values from baseline to 12 and 24 months and the change from 12 to 24 months. Statistical analysis found no difference in 15D between IBR and DR (*p* > 0.05).

For EORTC QLQ C30 BR-23, the Mann–Whitney U-test found a statistically significant difference in the change of sexual functioning scores between IBR and DR (*p* = 0.009). In both groups, sexual functioning was impaired from baseline to 24 months, but in the DR group the drop in scores was deeper and they did not recover as well as the IBR group during the follow-up. For arm symptoms there was a difference in the change from 12 to 24 months (*p* = 0.003): IBR patients’ arm symptoms diminished (score 15.2–10.4), but for DR they increased (17.5–19.4).

#### The comparison of scores

For the 15D, sleeping, discomfort and symptoms, and sexual functioning were the most impaired dimension scores within groups. There were no statistically significant differences between groups (*p* > 0.05) when comparing different surgical methods (Fig. 3).

For EORTC the only statistically significant difference in symptom scores was found for BR23 systemic therapy side effects at 12 months: IBR implant patients reported significantly less symptoms than abdominal flap patients (*p* = 0.034).

Later, at 24 months, patients reported symptoms with no difference between groups (all scores *p* > 0.05): Fatigue (IBR *p* = 0.748 and DR *p* = 0.884), Pain (IBR *p* = 0.482, DR *p* = 0.172) and Sleeping disturbances (IBR *p* = 0.983, DR *p* = 0.099). The functional scores between IBR and DR groups were not significantly different (*p* > 0.05) (Fig. 4).

### Complications

There were no life-threatening complications or deaths within either reconstruction group (class IV-V complications). In IBR groups, 18 patients (35.3%) encountered a complication. There was no total loss of flaps in this group, but 1 implant had to be removed due to infection. A class IIIb complication was recorded for 8 patients resulting from hematoma, infection or skin necrosis. Bilateral pulmonal embolism with no need for intensive care, was recorded for one implant patient. Puncture of an expander was recorded for one patient requiring more frequent fillings but having no effect on the final result.

In DR groups, 13 patients (31.7%) encountered a complication. A class IIIb complication was recorded for 7 patients. The only LAP-flap in this series was initially lost due to slowly fading circulation, 1 abdominal flap due to arterial thrombosis at 5 days post operation, and 1 LD patient’s implant was removed due to hematoma at the operation site. One patient developed a hernia to the abdominal area and had revision surgery with a mesh at the donor site (Table 2). Having a complication did not affect HRQoL, *p* > 0.05.

**Table 2:** Complications according Clavien-Dindo classification

	Clavien-Dindo I	Clavien-Dindo II	Clavien-Dindo IIIa	Clavien-Dindo IIIb
*Immediate reconstruction*				
LD				
*Recipient*	II		II	IIII
*Donor*				
Abdominal flap				
*Recipient*	I			III
*Donor*				
Implant	II	II	I	I
TMG				
*Delayed reconstruction*				
LD				
*Recipient*				I
*Donor*		I	I	
Abdominal flap				
*Recipient*			I	III
*Donor*	I			II
Implant				
Fat grafting				
Other		II		I

LD = latissimus dorsi flap, TMG = transverse musculocutaneus gracilis flap, Other = reduction, LAP

## Discussion

Our study describes breast cancer patients’ HRQoL with reconstructed breast. The healing process after reconstruction is variable, and patients cope with it within their personal capabilities. In the light of past studies, autologous methods seem to be more beneficial to patients [12, 13].

Our follow-up study with both generic and disease specific HRQoL instruments follows suggested, valid research strategies. However, the interpretation of our results is hampered by our rather small group sizes [5]. Unfortunately we had to stop our recruitment process a few times which resulted in a long study period. Even though we recruited more than 1000 breast cancer patients, it turned out at the analysis phase that the number of reconstruction patients was quite small. However, we were not able to continue the study any further. By years 2009 to 2011 the number of yearly IBR were around 100 patients per year at our unit. At the onset of our study, the readily available validated measuring tools were the generic 15D and the EORTC QLQ-30 BR 23 for breast cancer. Other available measuring tools seemed to be less suitable with higher ceiling-effects, like the EQ-5D or VAS [23].

HRQoL in both groups diminished after baseline and then started to rise, which could be seen as a natural course of the healing process. Breast reconstruction seems to be a favourable option as the overall HRQoL level proved to be at a reasonably high level (Figs. 1 and 2) and patients reported only little symptoms (Figs. 3 and 4) at 24 months. The mean time for DR was 20.5 months from baseline so all DR patients at 24 months had had good time to recover from initial cancer surgery and oncological treatments, but then had to start the recovery process all over again with breast reconstruction. Nevertheless, regarding timing of reconstruction, in line with past studies, the overall HRQoL did not differ between IBR and DR groups in our study [24].

A recent study identified risk factors for reconstruction complications: BMI over 30, asthma or other pulmonary disease, LD as a method and IBR as a whole [25]. There were slightly more complications in our study for LD patients in the IBR group, but the difference was not statistically significant. We found no correlation between patients having a complication and HRQoL and no differences between groups. A similar finding was reported in a study that compared IBR and DR patients having received radiation therapy [26].

Surgical methods, complications, or the length of stay at hospital did not affect HRQoL in our study. The symptoms, that patients reported are in line with past studies [27]. Fatigue, pain and sleeping disturbances are commonly reported symptoms regardless of the surgical method.

## Conclusion

Autologous reconstruction produces good HRQoL. Different autologous methods can be considered to be equally acceptable. Immediate or delayed timing for breast reconstruction should be tailored according to the patient’s individual need while both strategies produce good HRQoL.
